# Comparison of 2-year outcomes between primary and secondary prophylactic use of defibrillators in patients with coronary artery disease: A prospective propensity score–matched analysis from the Nippon Storm Study

**DOI:** 10.1016/j.hroo.2020.12.020

**Published:** 2021-01-07

**Authors:** Yusuke Kondo, Takashi Noda, Yasunori Sato, Marehiko Ueda, Takashi Nitta, Yoshifusa Aizawa, Tohru Ohe, Takashi Kurita

**Affiliations:** ∗ Department of Cardiovascular Medicine, Chiba University Graduate School of Medicine, Chiba, Japan; † Department of Cardiovascular Medicine, National Cerebral and Cardiovascular Center, Osaka, Japan; ‡ Department of Preventive Medicine and Public Health, Keio University School of Medicine, Tokyo, Japan; § Department of Cardiology, Eastern Chiba Medical Center, Chiba, Japan; ‖ Department of Cardiovascular Surgery, Nippon Medical School, Tokyo, Japan; ¶ Department of Research and Development, Tachikawa Medical Center, Niigata, Japan; # Okayama City Hospital, Okayama, Japan; ∗∗ Department of Internal Medicine, Faculty of Medicine, Kindai University, Osaka, Japan

**Keywords:** Coronary artery disease, Implantable cardioverter-defibrillator, Nippon Storm Study, Primary prophylaxis, Secondary prophylaxis

## Abstract

**Background:**

The Nippon Storm Study was a prospective observational study designed to gather clinical data on implantable cardioverter-defibrillator (ICD) therapy in Japanese patients.

**Objective:**

The purpose of this subanalysis was to compare the incidence of ICD therapy in patients with left ventricular dysfunction owing to coronary artery disease (CAD) for primary and secondary prophylaxis of sudden cardiac death.

**Methods:**

We analyzed data of 493 patients with CAD and ICDs (men, 87%; age, 68 ± 10 years; left ventricular ejection fraction, 36% ± 13%; primary prophylaxis, 36%). All patients were followed up for at least 2 years. Propensity score matching was used to select patient subgroups for comparison: 133 patients with ICD for primary prophylaxis and 133 with ICD for secondary indications.

**Results:**

There were no significant differences between primary and secondary prophylaxis groups with respect to the incidence of appropriate ICD therapy within 2 years (0.153 vs 0.239; hazard ratio, 1.565 [95% confidence interval (CI), 0.898–2.727]; *P* = .114). Two-year electrical storm risks were 3.3% and 9.6% with HR = 3.236 (95% CI, 1.058–9.896; *P* = .039) in patients with primary and secondary prophylaxis, respectively.

**Conclusion:**

The incidence of ICD therapy received by patients with CAD for primary and secondary prophylaxis was not significantly different based on our propensity score–matched analysis. However, secondary-prophylaxis ICD therapy seems to be associated with a significantly higher risk for electrical storm than primary-prophylaxis ICD therapy.

## Graphical abstract

**Figure:**
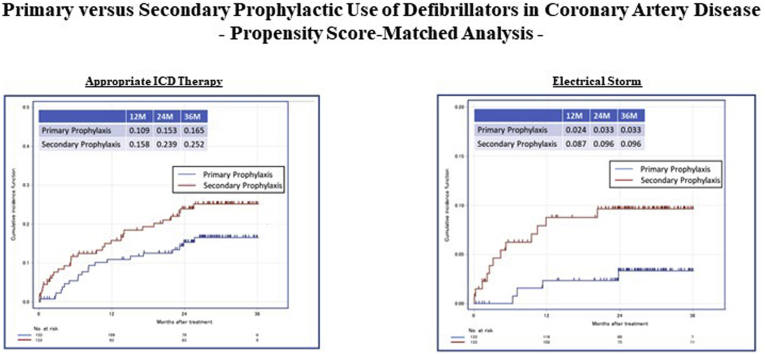

## Introduction

Treatment with implantable cardioverter-defibrillators (ICDs) is used worldwide to prevent sudden cardiac death (SCD) in patients with ischemic cardiomyopathy who are at risk of fatal arrhythmic events.^1^, ^2^, ^3^ Nevertheless, there have been no large-scale studies on prophylactic ICD therapy in Japan. Improvements in revascularization techniques, such as percutaneous coronary intervention (PCI) and coronary artery bypass grafts, have contributed to decreased cardiovascular mortality in patients with coronary artery disease (CAD) over the past few decades.^4^^,^^5^ The Nippon Storm Study was a prospective observational study designed to gather clinical data from Japanese patients receiving ICD therapy.^6^^,^^7^ In total, 1570 patients were enrolled at 48 ICD centers in Japan. We focused on 1274 patients with structural heart disease, including 493 (38.6%) patients with CAD. We aimed to compare the incidence of ICD therapies for primary and secondary prophylaxis of SCD in patients with left ventricular (LV) dysfunction attributable to CAD.

## Methods

### Registration

The Nippon Storm Study design details have been previously published.^6^^,^^7^ Briefly, the study was organized by the Japanese Heart Rhythm Society and the Japanese Society of Electrocardiology. Data collection concerning the registration of new transvenous ICD patients began in October 2010 and data accumulation for the registry was terminated in July 2012. Patient registration was conducted via a website at 48 Japanese ICD centers, and the Japanese Heart Rhythm Society collected patient data obtained from physicians. The indications and purposes of implantation were determined by the attending cardiologists at each center based on the Japanese Circulation Society (JCS) Guidelines for Non-Pharmacotherapy of Cardiac Arrhythmias (JCS 2011) for implantation of an ICD.^8^ The present study was approved by the Institutional Review Board’s Ethics Committee at Kindai University (reference number: 10-04). Each patient completed a written informed consent form. This study was conducted according to the principles of the Helsinki Declaration, as revised in 2013.

### ICD programming

ICDs were programmed at the physician’s discretion. We used the following discrimination algorithms: PR Logic and Wavelet (Medtronic, Minneapolis, MN), Rhythm ID (Boston Scientific, Marlborough, MA), and Morphology Discrimination plus AV Rate Branch (St. Jude Medical, St. Paul, MN). The ventricular fibrillation (VF) zone was ≥180 to 200 beats per minute with at least 1 train of antitachycardia pacing (ATP) before the shock, and the ventricular tachycardia (VT) zone was ≥140 to 160 beats per minute with at least 3 trains of ATP before the shock, which could be modified according to patient background and could be programmed for the fast VT zone if needed.

### Follow-up

For precise follow-up, we constructed a new tracking system called “Chaser,” which was intended to minimize the loss of follow-up data. Data regarding ICD interventions were sent at a maximum interval of 6 months to the office of the Japanese Heart Rhythm Society through the website. The ICD interventions were classified into ATP and shock therapies. An electrical storm (ES) was defined as occurrence of at least 3 separate episodes of VT/VF within a 24-hour period.^9^

### Statistical analysis

For the baseline characteristics, summary statistics were expressed as frequencies and proportions for categorical data, and as means and standard deviations (SD) for continuous variables. We compared patient characteristics using χ^2^ or Fisher exact test for categorical outcomes and using *t* test for continuous variables.

Treatment selection was considered to be influenced not only by the characteristics of patients, because the baseline characteristics of subjects receiving a particular treatment often differ systematically from those of subjects receiving an alternative treatment. This is an important issue when estimating the effect of treatments or exposures on outcomes using observational data. One approach to reduce or eliminate the effect of treatment selection bias and confounding effects is to use propensity score matching, which allows the design and analysis of an observational (nonrandomized) study to mimic some of the characteristics of a randomized controlled trial. Patient selection was performed by employing the propensity score matching method with a Greedy 5-to-1 digit-matching algorithm for clinical factors, ie, age, sex, cardiac resynchronization therapy with defibrillator, LV ejection fraction (LVEF). After all propensity score matches had been performed, we compared baseline covariates between the 2 groups.

The main purpose of this study was to compare the incidence of ICD therapies between primary and secondary prophylaxis groups of patients with CAD. For time-to-event outcomes, the Kaplan-Meier method was used to estimate the overall survival and cardiovascular survival by each group, and the differences in survival between groups were analyzed with the log-rank test. The hazard ratios (HRs) and 95% confidence intervals (CIs) were calculated using the Cox proportional hazards model. The cumulative incidences of ICD therapies were calculated using competing risk analysis, because death is a competing risk for loss to follow-up. The competing risk analyses were performed using the Fine-Gray generalization of the proportional hazards model accounting for death as a competing risk. Fine-Gray uses the subdistribution hazard to model cumulative incidence. All comparisons were planned, and the tests were 2-sided. A *P* value < .05 was considered statistically significant. All statistical analyses were performed using SAS software version 9.4 (SAS Institute, Cary, NC).

## Results

### Study cohort

We focused on 493 patients with CAD out of 1274 patients who had structural heart disease. After propensity matching, the analysis was restricted to 266 patients: 133 (50%) with ICDs for primary prophylaxis and 133 (50%) with ICDs for secondary prophylaxis.

### Baseline patient characteristics

In the overall cohort of patients with CAD, the mean age was 68 ±10 years, and 87% of patients were men (Table 1). Most of the baseline characteristics were differently distributed between the primary and secondary prophylaxis groups (Table 2). Patients in the primary prophylaxis group were older, were more likely to receive cardiac resynchronization therapy with a defibrillator, had more severe heart failure, and were less likely to receive class III antiarrhythmic drugs.

**Table 1:** Baseline characteristics of patients with coronary artery disease

	Ischemic heart disease (N = 493)
Sex, male	429 (87.0%)
Age, yeas	67.8 ± 10.0
Primary prophylaxis	178 (36.1%)
Single-chamber ICD	85 (17.2%)
CRTD	150 (30.4%)
NYHA functional class III or IV	143 (29.0%)
LVEF	36.0 ± 13.4
BNP, pg/mL	500.6 ± 744.4
Hb, g/dL	12.3 ± 1.9
HT	278 (56.4%)
DL	225 (45.6%)
DM	205 (41.5%)
Stroke	47 (9.5%)
HU	75 (15.2%)
PAD	22 (4.5%)
3VD or LMT	136 (27.6%)
Diuretics	308 (62.4)
β-blocker	316 (64.1%)
ACE-I or ARB	323 (65.5%)
Antiplatelet agent	400 (81.1%)
Anticoagulant agent	140 (28.3%)
Class III antiarrhythmic drugs	254 (51.5%)

Values are presented as mean ± SD or n (%).

ACE-I = angiotensin-converting enzyme inhibitors; ARB = angiotensin II receptor; BNP = brain natriuretic peptide; CRTD = cardiac resynchronization therapy with defibrillator; DL = dyslipidemia; DM = diabetes mellitus; Hb = hemoglobin; HT = hypertension; HU = hyperuricemia; ICD = implantable cardioverter-defibrillator; LMT = left main trunk; LVEF = left ventricular ejection fraction; NYHA = New York Heart Association; PAD = peripheral arterial disease; 3VD = 3-vessel disease.

**Table 2:** Baseline characteristics of unmatched and propensity core–matched cohort

	Unmatched cohort			Matched cohort		
	Primary prophylaxis (n = 178)	Secondary prophylaxis (n = 315)	*P* value	Primary prophylaxis (n = 133)	Secondary prophylaxis (n = 133)	*P* value
Male	158 (88.7%)	271 (86.0%)	0.3860	117 (88.0%)	118 (88.7%)	0.8485
Age, years	70.0 ± 8.7	66.6 ± 11.1	<0.001	69.1 ± 8.5	70.6 ± 8.8	0.0767
Single-chamber ICD	28 (15.7%)	57(18.1%)	0.5043	25 (18.7%)	22 (16.5%)	0.6269
CRTD	95 (53.3%)	55 (17.4%)	<0.001	50 (37.6%)	46 (34.5%)	0.6096
NYHA functional class III or IV	76 (42.6%)	67 (21.2%)	<0.001	44 (33.1%)	44 (33.1%)	1.0000
LVEF, %	30.5 ± 10.8	39.1 ± 13.7	<0.001	31.9 ± 11.4	33.3 ± 11.4	0.1595
BNP, pg/mL	513.1 ± 570.7	493.1 ± 831.1	0.3859	476.9 ± 518.6	546 ± 817	0.2222
Hb, g/dL	12.4 ± 2.0	12.2 ± 1.9	0.0778	12.7 ± 2.0	12.1 ± 2.0	0.0079
HT	110 (61.7%)	168 (53.3%)	0.0687	83 (62.4%)	88 (66.2%)	0.5223
DL	81 (45.5%)	144 (45.7%)	0.9644	66 (49.6%)	63 (47.3%)	0.7128
DM	79 (44.3%)	126 (4.0.0%)	0.3430	55 (41.4%)	59 (44.4%)	0.6202
Stroke	18 (10.1%)	29 (9.2%)	0.7422	13 (9.8%)	10 (7.5%)	0.5128
HU	32 (17.9%)	43 (13.6%)	0.1989	26 (19.5%)	27 (20.3%)	0.8780
PAD	6 (3.3%)	16 (5.1%)	0.3775	3 (2.3%)	8 (6.0%)	0.1236
3VD or LMT	56 (31.4%)	80 (25.3%)	0.1479	39 (29.3%)	37 (27.8%)	0.7860
Diuretic agent	130 (73.0%)	178 (56.5%)	<0.001	98 (73.6%)	87 (65.4%)	0.1428
β-blocker	120 (67.4%)	186 (59.0%)	0.2878	89 (66.9%)	84 (63.2%)	0.5203
ACE-I/ARB	124 (69.6%)	199 (63.1%)	0.1455	93 (69.9%)	78 (58.6%)	0.0549
Antiplatelet agent	147 (82.5%)	253 (80.3%)	0.5366	109 (81.9%)	107 (80.4%)	0.7536
Anticoagulant agent	53 (29.7%)	87 (27.6%)	0.6101	45 (33.8%)	35 (26.3%)	0.1812
Class III antiarrhythmic drug	57 (32.0%)	197 (62.5%)	<0.001	42 (31.5%)	90 (67.6%)	<0.001

Values are presented as mean ± SD or n (%).

ACE-I = angiotensin-converting enzyme inhibitors; ARB = angiotensin II receptor; BNP = brain natriuretic peptide; CRTD = cardiac resynchronization therapy with defibrillator; DL = dyslipidemia; DM = diabetes mellitus; Hb = hemoglobin; HT = hypertension; HU = hyperuricemia; ICD = implantable cardioverter-defibrillator; LMT = left main trunk; LVEF = left ventricular ejection fraction; NYHA = New York Heart Association; PAD = peripheral arterial disease; 3VD = 3-vessel disease.

After propensity score matching, the baseline characteristics considered for propensity score calculation, except for class III antiarrhythmic drugs treatment, were almost equally distributed between the 2 study groups.

### ICD therapy

**Figure 1:**
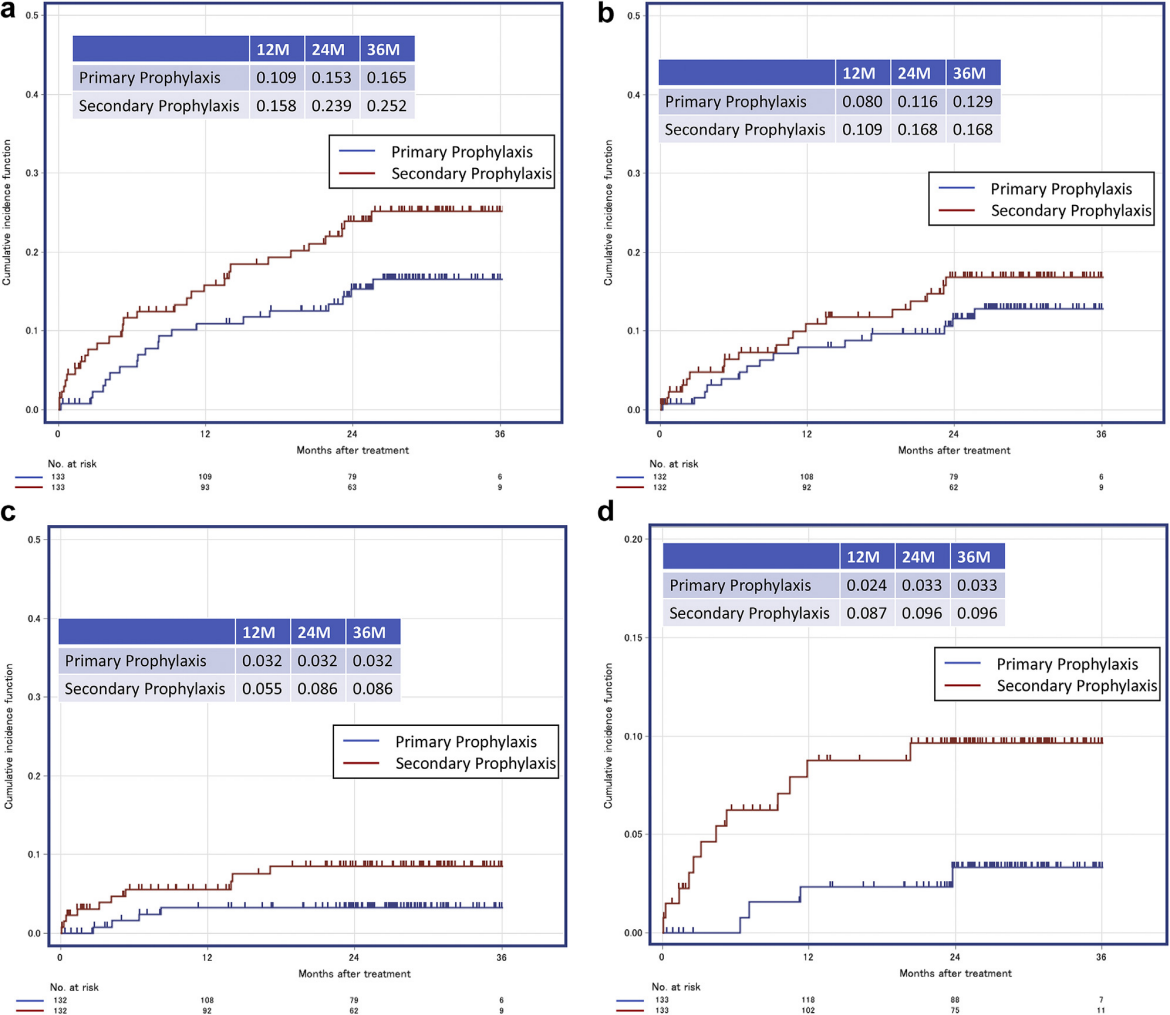
Incidence of appropriate implantable cardioverter-defibrillator therapy (**a**), appropriate antitachycardia pacing therapy (**b**), appropriate shock therapy (**c**), and electrical storm (**d**) in the propensity score–matched cohorts of patients with the primary and secondary prophylaxis.

**Figure 2:**
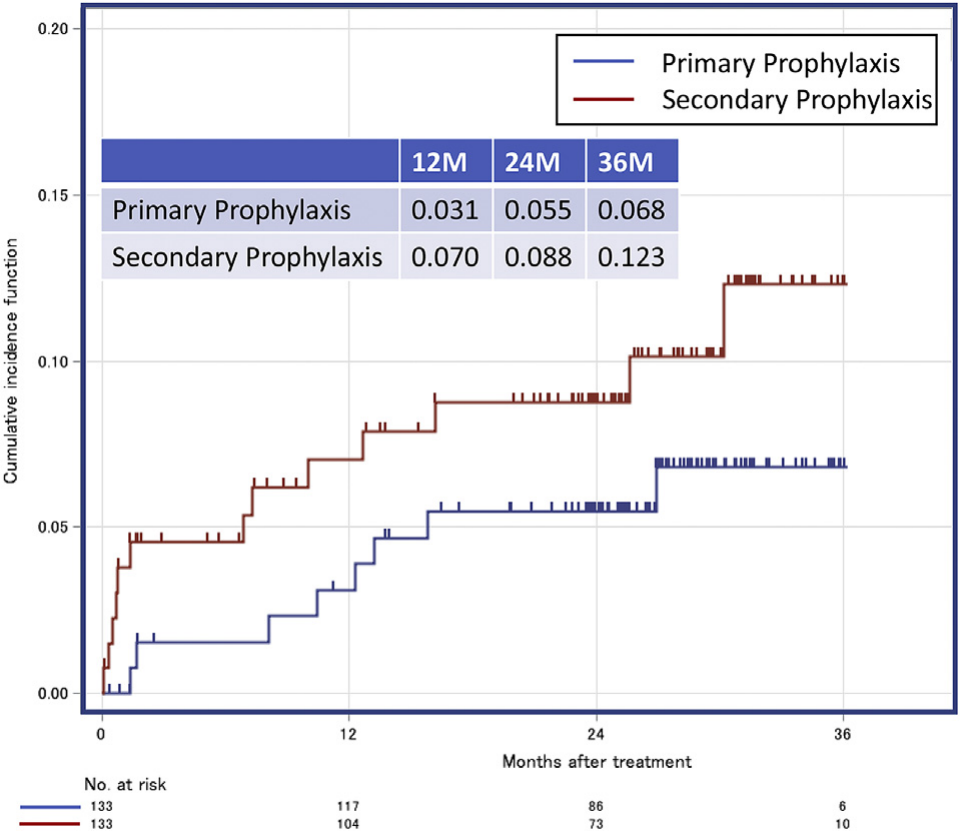
Incidence of inappropriate implantable cardioverter-defibrillator therapy in the propensity score–matched cohorts of patients with the primary and secondary prophylaxis.

In the matched cohort, the 2-year appropriate ICD therapy risks were 15.3% and 23.9% in the primary and secondary prophylaxis groups, respectively (HR = 1.565 [95% CI, 0.898–2.727; *P* = .114]) (Figure 1). Two-year appropriate ATP risks were 11.6% and 16.8% in the primary and secondary prophylaxis groups, respectively (HR = 1.302 [95% CI, 0.670–2.529; *P* = .436]), and appropriate shock therapy risks were 3.2% and 8.6% in the primary and secondary prophylaxis groups, respectively (HR = 2.700 [95% CI, 0.850–8.575; *P* = .092]). Two-year ES risks were 3.3% and 9.6% with HR = 3.236 (95% CI, 1.058–9.896; *P* = .039) in the primary and secondary prophylaxis groups, respectively. The 2-year inappropriate ICD therapy risks were 5.5% and 8.8% in the primary and secondary prophylaxis groups, respectively (HR = 1.749 [95% CI, 0.733–4.174; *P* = .208]) (Figure 2).

### Survival

**Figure 3:**
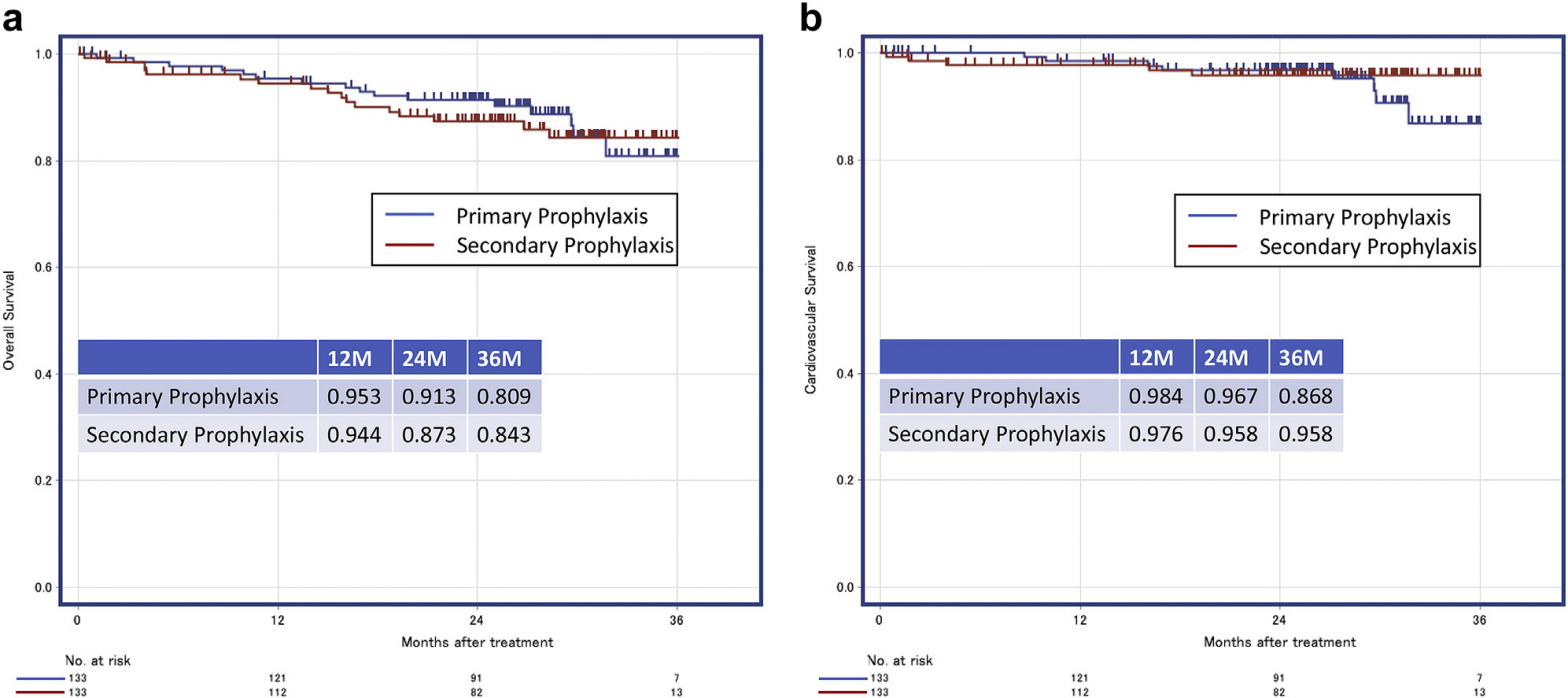
Event-free overall survival (**a**) and cardiovascular survival (**b**) in the propensity score–matched cohorts of patients with the primary and secondary prophylaxis.

In this matched cohort, 33 (12.4%) all-cause deaths and 13 (4.9 %) cardiovascular deaths occurred over a median follow-up of 791 days (interquartile range, 621–937 days). The 2-year overall survival rates were 91.3% and 87.3% in the primary and secondary prophylaxis groups, respectively (HR = 0.943 [95% CI, 0.728–1.221; *P* = .655]) (Figure 3). The 2-year cardiovascular survival rates were 96.7% and 95.8% in the primary and secondary prophylaxis groups, respectively (HR = 1.567 [95% CI, 0.505–4.857; *P* = .436]).

## Discussion

The major findings of the present study in patients with CAD in Japan were as follows: (1) appropriate use of ICD therapy was comparable in the primary and secondary prophylaxis patient groups; (2) the risk for ES was significantly higher in patients with secondary prophylaxis than in those with primary prophylaxis; and (3) there were no significant differences in mortality or cardiovascular survival rates between the primary and secondary prophylaxis groups.

### Benefits of prophylactic ICD therapy

Previous studies have demonstrated the benefits of ICD therapy in survivors of VF or symptomatic sustained VT.^10^, ^11^, ^12^ Based on recent findings, the DANISH study may now be considered somewhat controversial with respect to the efficacy of primary prophylactic ICD implantation in patients with nonischemic systolic heart failure.^13^ In the initial earlier period of primary PCI, most patients with acute myocardial infarction (MI) could receive early coronary revascularization; however, there has been controversy regarding the efficacy of ICD for primary prophylaxis of major adverse cardiovascular events in patients with CAD.

The Multicenter Automatic Deﬁbrillator Trial II (MADIT II) demonstrated that postinfarction patients with LVEF of ≤30% without the need for invasive electrophysiological testing beneﬁted from prophylactic therapy with ICDs.^2^ During an average follow-up of 20 months, the mortality rates were 19.8% and 14.2% in the conventional and defibrillator groups, respectively. There was a 31% reduction in the risk of all-cause mortality in patients randomized to an ICD group compared with patients receiving conventional optimal therapy with high usage of beta blockers, angiotensin-converting enzyme inhibitors / angiotensin II receptor blockers, and statins. Moreover, treatment with an ICD was associated with a significant reduction in the risk of death during the early phase of the extended follow-up period (0 through 4 years: HR = 0.61 [95% CI, 0.50–0.76]; *P* < .001) and with continued life-saving benefit during the late phase of follow-up (5 through 8 years: HR = 0.74 [95% CI, 0.57–0.96]; *P* = .02) in MADIT II.^3^

There have been several reports of lower SCD rates in patients meeting MADIT II criteria (postinfarction with ≤30% LVEF) but without an ICD in Japan.^14^^,^^15^ A low incidence of SCD was recently reported in patients with MI who underwent primary PCI in Japan.^16^ The Chronic Heart Failure Analysis and Registry in the Tohoku District-2 (CHART-2) study revealed that Japanese patients eligible for prophylactic ICD implantation did not always receive this therapy in real-world practice.^17^ ICD remains underused in a substantial proportion of patients who meet the guidelines for primary prophylaxis in Japan, possibly because of the invasive nature of the procedure in a population of patients who have not yet experienced prior life-threatening arrhythmias and because of cost considerations. Various factors such as age, renal failure, liver failure, cancer, and failure to receive a referral from a general physician or from a cardiologist to an electrophysiologist are likely to explain underuse of ICD prophylactic implantation.

In this subanalysis of the Nippon Storm Study, the risk of SCD owing to VT/VF in patients with LV dysfunction and CAD were comparably high in both primary and secondary prophylaxis groups. While the benefit of secondary prophylactic ICD therapy was firmly established, ICD therapy is also specifically recommended for primary prophylaxis to reduce total mortality through a reduction in SCD in patients with LV dysfunction (LVEF ≤35%). Essentially, evaluation should be deferred until at least 3 months after revascularization procedures and, more strictly, until at least 40 days after acute MI to allow adequate time for recovery of myocardial function. The wearable cardioverter-defibrillator therapy is considered in patients with high risk of SCD in the early post-MI phase because underuse of ICD remains a major problem in clinical practice in Japan.

### Electrical storm

ES is a devastating, life-threatening event that is becoming more commonly observed in clinical practice. The present subanalysis of the Nippon Storm Study that enrolled patients with an ICD revealed that the risk for ES was significantly higher in the patients with secondary prophylaxis than in those with primary prophylaxis (HR = 3.236; 95% CI, 1.058–9.896; *P* = .039).

The incidence of ES is steadily rising worldwide along with the number of patients with an ICD.^18^ Previous studies have reported that approximately 4%–7% of patients with ICDs implanted for primary prevention and 10%–58% of those with ICDs implanted for secondary prevention would experience an episode of ES at some point after implantation.^19^^,^^20^ An increased susceptibility to VT/VF represents a proarrhythmic burden in patients with secondary prophylaxis. ES may be a result of interplay between preexisting pathologic conditions that create a vulnerable substrate and patient-specific initiating factors, such as worsening of heart failure, emotional stress, alcohol excess, and myocardial ischemia. Nedios and colleagues^20^ showed that secondary prophylaxis and prior ICD therapies remain the most significant predisposing factors to ES. A subanalysis of the MADIT-II trial suggested that patients with LV dysfunction and CAD who experience ES were at a markedly increased risk for subsequent death.^21^ Once a patient had ES, they were at a significantly higher risk of dying than patients with isolated episodes of VT/VF or those with no events. Prompt initiation of aggressive treatment, especially radiofrequency catheter ablation, might be considered in the acute management of drug-refractory ES.

### Study limitations

This study had several limitations. First, its prospective observational design and multicenter registry resulted in a lack of randomization. Moreover, the sample size was relatively small and underpowered, in particular, the propensity score matched analysis; therefore, there was a risk of possible hidden bias. Second, a relatively low heart rate programming for VT/VF detection in our series may have increased the incidence of ES through providing unnecessary ICD therapy for self-terminating VT/VF. Nevertheless, this programming trend was observed primarily in patients with secondary prophylaxis, and a detection interval was determined based on the lowest rate of clinical VT in each patient. In patients with primary prophylaxis, higher detection rates and longer duration intervals were used compared with patients with secondary prophylaxis. Any difference in programming detection zones and discrimination algorithms between the groups would have affected the outcome. Third, despite propensity-matched scoring, there was a major difference in the percentage of patients who received class III antiarrhythmics, with many more such patients in the secondary prophylactic group. Therefore, we compared a group of more unwell patients who had a higher risk of VT/VF and a much more potent antiarrhythmic drug regimen with a group of less unwell patients who, a priori, would be less likely to have VT/VF. Further large population studies to address this issue are needed.

## Conclusion

Appropriate ICD therapy is commonly applied at comparable rates for either primary or secondary prophylaxis in patients with CAD-related LV dysfunction. However, the risk for ES is significantly higher in patients with secondary prophylaxis than in those with primary prophylaxis.

## Acknowledgments

We gratefully acknowledge all 48 Japanese implantable cardiac shock device centers involved in this study; the office of the Japanese Heart Rhythm Society, especially Ms Yoko Sato; and Ms Shoko Narumi and Mr Jin Ono for data collection.

## Funding Sources

No financial support was received for this study from any specific company, except the Japan Arrhythmia Device Industry Association. This study was partially supported by JSPS KAKENHI Grant Number JP085700004.

## Disclosures

The authors have no conflicts to disclose.

## Authorship

All authors attest they meet the current ICMJE criteria for authorship.

## Patient consent

Each patient completed a written informed consent form.

## Ethics statement

The present study was approved by the Institutional Review Board's Ethics Committee at Kindai University (reference number: 10-04) and was conducted according to the principles of the Helsinki Declaration, as revised in 2013.
